# Defects in Glycosylation Impair Satellite Stem Cell Function and Niche Composition in the Muscles of the Dystrophic Large^myd^ Mouse

**DOI:** 10.1002/stem.1197

**Published:** 2012-09-20

**Authors:** Jacob Ross, Abigail Benn, Jacqueline Jonuschies, Luisa Boldrin, Francesco Muntoni, Jane E Hewitt, Susan C Brown, Jennifer E Morgan

**Affiliations:** a Dubowitz Neuromuscular Centre, Institute of Child Health, University College LondonLondon, United Kingdom; b Institute of Genetics, School of Biology, Queen's Medical Centre, University of NottinghamNottingham, United Kingdom; c Comparative Biomedical Sciences, Royal Veterinary College, University of LondonLondon, United Kingdom

**Keywords:** Skeletal muscle satellite cells, Adult stem cells, α-Dystroglycanopathies, Congenital muscular dystrophy, Extracellular matrix, Muscle regeneration

## Abstract

The dystrophin-associated glycoprotein complex (DGC) is found at the muscle fiber sarcolemma and forms an essential structural link between the basal lamina and internal cytoskeleton. In a set of muscular dystrophies known as the dystroglycanopathies, hypoglycosylation of the DGC component α-dystroglycan results in reduced binding to basal lamina components, a loss in structural stability, and repeated cycles of muscle fiber degeneration and regeneration. The satellite cells are the key stem cells responsible for muscle repair and reside between the basal lamina and sarcolemma. In this study, we aimed to determine whether pathological changes associated with the dystroglycanopathies affect satellite cell function. In the Large^myd^ mouse dystroglycanopathy model, satellite cells are present in significantly greater numbers but display reduced proliferation on their native muscle fibers in vitro, compared with wild type. However, when removed from their fiber, proliferation in culture is restored to that of wild type. Immunohistochemical analysis of Large^myd^ muscle reveals alterations to the basal lamina and interstitium, including marked disorganization of laminin, upregulation of fibronectin and collagens. Proliferation and differentiation of wild-type satellite cells is impaired when cultured on substrates such as collagen and fibronectin, compared with laminins. When engrafted into irradiated tibialis anterior muscles of *mdx*-nude mice, wild-type satellite cells expanded on laminin contribute significantly more to muscle regeneration than those expanded on fibronectin. These results suggest that defects in α-dystroglycan glycosylation are associated with an alteration in the satellite cell niche, and that regenerative potential in the dystroglycanopathies may be perturbed.

## INTRODUCTION

The dystroglycanopathies are a set of muscular dystrophies characterized by defects in the glycosylation of α-dystroglycan, a component of the dystrophin-associated glycoprotein complex [1–3]. This multiprotein complex is present at the sarcolemma and links the external extracellular matrix (ECM) to the internal actin cytoskeleton of muscle fibers. Components in the basal lamina portion of the ECM, such as laminin-211, agrin, and perlecan, interact with the heavily glycosylated α-dystroglycan on the external leaf of the sarcolemma [4–6]; this is itself associated with the trans-sarcolemmal β-dystroglycan, which binds dystrophin on the subsarcolemmal side [7]. Dystrophin associates with actin in the cytoskeleton, and the whole complex provides a structural link to ensure sarcolemmal stability and the presumed transmission/dissipation of forces, as well as roles in cell signaling [8].

The dystroglycanopathies are associated with mutations in nine currently known genes [3]. Only one case has been reported so far with a pathogenic mutation in the dystroglycan (DAG1) gene itself [9], which therefore represents a primary dystroglycanopathy; the other eight genes encode proteins with confirmed or putative roles in the glycosylation of α-dystroglycan and are therefore secondary dystroglycanopathies. Among these are the glycosyltransferases that transfer glycan structures onto α-dystroglycan: protein *O*-mannosyltransferases 1 and 2 (POMT1 and 2) [10]; protein *O*-linked-mannose β-1,2-*N*-acetylglucosaminyltransferase 1 (POMGnT1) [11], and Large [12]; and the putative glycosyltransferases fukutin [13] and fukutin-related protein [1]. Two recently identified genes that encode for dolichol-phosphate-mannose synthase (DPM) subunits DPM2 [14] and DPM3 [15] have also been found to be defective in patients with a dystroglycanopathy phenotype, consistent with the role of the DPM complex in the synthesis of glycan precursors for the *O*-mannosyl glycosylation of α-dystroglycan.

The presence of *O*-mannosylated glycans on α-dystroglycan is known to be essential for mediating its binding to extracellular ligands in the basal lamina, and their loss is thought to be central to the pathogenesis of the dystroglycanopathies [2, 3, 16]. Mutations in POMT1, POMT2, POMGnT1, Fukutin, Fukutin-related protein, and Large are associated with an uncommonly wide clinical spectrum. At the most severe end are patients with the congenital muscular dystrophy variants Walker-Warburg syndrome and muscle-eye-brain (MEB) disease, which present with dystrophic muscle pathology, structural brain and eye abnormalities, mental retardation, and often a heavily shortened life-span; at the mild end, there are patients with limb girdle muscular dystrophy 2I, which manifest with a mild muscle pathology and no eye or structural brain involvement [16].

Satellite cells are the principal muscle stem cell, residing between the sarcolemma of muscle fibers and the surrounding basal lamina [17–21]. Under normal conditions, satellite cells are quiescent, expressing the paired-box transcription factor Pax7 [22, 23]. In response to damage, they become activated, re-enter the cell cycle, and proliferate extensively, expressing myogenic regulatory factors (MRFs) such as MyoD, MRF4, and Myf5 [24]. Eventually, they give rise to myoblast progeny that express myogenin (MyoG), whereby they exit the cell cycle and fuse with each other to make new muscle fibers or with existing fibers to repair the damage [25, 26]. A set of reserve cells do not undergo fusion but instead maintain Pax7 expression and enter quiescence once more, to repopulate the niche with self-renewed satellite cells [27, 28]. Basal lamina components in the niche (the microenvironment that surrounds stem cells) and other extrinsic signals are known to govern satellite cell behavior; among them, laminin-211, the major muscle isoform of this class of proteins, is known to be particularly important [29–31].

In the dystroglycanopathies, muscle fibers undergo repetitive cycles of degeneration and regeneration due to the presence of a structurally compromised sarcolemma/basement membrane. While satellite cell-mediated repair of this damage may be successful in the early stages, previous studies in other muscular dystrophies suggest that a cell-intrinsic reduction in the regenerative capacity of these cells eventually becomes a limiting factor, and muscle fibers are gradually replaced by connective tissue [32–34]. Additionally, alterations to the basal lamina microenvironment may further impinge upon satellite cell activity. Dystroglycan has been shown to organize laminin and other basal lamina components into networks and as such has been attributed with a primary role in basement membrane deposition [6, 35]. Given the well-documented mitogenic effect of laminin on myoblasts [30, 31, 36], any disruption in its ability to bind to laminin might be expected to influence the satellite cell niche. Indeed, in support of this, previous satellite cell/myoblast studies in dystrophin-deficient Duchenne muscular dystrophy (DMD) patients point to either elevated or decreased numbers of satellite cells in muscle, depending on the report and method used [37, 38]. DMD myoblasts also display reduced proliferation and differentiation, and POMGnT1-null mouse myoblasts reduced proliferation, in vitro [39, 40].

In this study, we use the Large^myd^ mouse, a dystroglycanopathy model with a pathology resembling MEB disease, to investigate satellite cell function. Large^myd^^−/−^ but not Large^myd^^+/−^ mice have severe muscle pathology, together with eye and structural brain defects [41–44]. We report an elevation in satellite cell number on freshly isolated single muscle fibers of Large^myd^ mice, together with impaired proliferation, compared with those of wild-type. However, proliferative capacity is restored to that of wild-type when satellite cells are removed from their native fibers and subsequently cultured. Immunohistochemical analysis reveals satellite cells in contact with fibronectin and laminin. However, in Large^myd−/−^ muscle there is a marked disorganization of laminin and occasionally a discontinuity in its contact with satellite cells. Other pathological hallmarks here include the upregulation of fibronectin in the basal lamina compared with wild-type muscle and associated fibrosis. The importance of basal lamina components in directing satellite cell/myoblast behavior was investigated in vitro, with laminins, but not fibronectin or collagen, enhancing both proliferation and differentiation. Furthermore, cells expanded on fibronectin have limited regenerative capacity in vivo, compared with satellite cells cultured on laminin-111. These results highlight the importance of the basal lamina in regulating satellite cell activity and suggest that potential alterations to this niche in disease may be a factor in satellite cell dysregulation.

## MATERIALS AND METHODS

### Single Fiber and Primary Satellite Cell Isolation

Mice were bred, and all experiments were carried out under Home Office License at the University College London Institute of Child Health, the Royal Veterinary College London, or the University of Nottingham in accordance with the Animals (Scientific Procedures) Act 1986. Extensor digitorum longus (EDL) muscles were carefully dissected from C57BL/6 mice (3–6 months old) or from Large^myd^ mouse litters (2–3 months old), so as to leave tendons intact. Studies between Large^myd^ and wild-type mice were from animals born in the same litters (from heterozygous pairings), as much as was feasible. Single muscle fibers were isolated as described previously [27], with minor modification. Briefly, muscle was digested in 2 mg/ml collagenase I (Sigma Aldrich, Dorset, U.K., http://www.sigmaaldrich.com) in Dulbecco's modified Eagle's medium (DMEM; Invitrogen, Paisley, U.K., http://www.invitrogen.com) for 130 minutes and titurated with a wide-bored pipette to release single fibers. For isolation of primary satellite cells, freshly isolated fibers were stripped of their basal lamina using 19-gauge needle and syringe and filtered through 40 μm cell sieves to remove debris.

### Culture of Muscle Fibers

All cultures of single fibers were incubated at 37°C, with 5% CO_2_ and atmospheric O_2_ concentrations. Fibers were cultured in DMEM + 10% horse serum (HS; Invitrogen) + 0.5% chicken embryo extract (CEE; Seralab, West Sussex, U.K., http://www.seralab.co.uk) + 2% l-glutamine (Gibco, Grand Island, NY, http://www.invitrogen.com) + 1% penicillin/streptomycin (Gibco), in suspension for up to 4 days (20 fibers in 8 ml), before staining for satellite cell and/or basal lamina markers (described below).

### Culture of Primary Satellite Cells

#### Clonal Analysis

Cells were diluted and plated in 96-well plates, such that approximately one-third of wells received a cell. It was assumed that resultant colonies originated from a single cell. Cultures were grown in proliferation medium (20% HS, 2% CEE) for 7 days or with a switch to differentiation medium (10% HS, 1% CEE) for a further 3 days. Laminin-111 (Sigma Aldrich) was used as standard substrate (5 μg/cm^2^ in minimal volume for coverage). For further characterization of substrates, the following were also used at the same density: mouse fibronectin (Biopur AG, Bubendorf, Switzerland, http://www.biopur.com), mouse collagen IV (Amsbio, Abingdon, U.K., http://www.amsbio.com), gelatin (Sigma Aldrich), Matrigel (BD Biosciences, Oxford, U.K., http://www.bdbiosciences.com), human laminin-211 (Millipore, Watford, U.K., http://www.millipore.com), or uncoated tissue culture plastic. At the appropriate time points, cells were fixed in 4% paraformaldehyde for 10 minutes, washed twice, stained with 0.04% trypan blue for visualization, and washed three times. Manual counts were made of total number of cells per colony over an entire well for proliferation studies, and number of fusion events for differentiation studies (myotubes containing two or more nuclei).

#### Expansion of Satellite Cells Prior to Engraftment

Satellite cells from *Myf5*^nlacZ/+^ mice (Expressing nLacZ, driven by the promoter of the satellite cell-specific marker *Myf5*) were grown in proliferation medium + 100 ng/ml fibroblast growth factor-2 (FGF2, Invitrogen), for 9 days at 6 cells per cm2 in 5% oxygen (previously shown to enhance regeneration postengraftment; D. Briggs, personal communication). Substrates were laminin-111 or fibronectin.

### Engraftments of Satellite Cells and Muscle Harvesting

4-week old *mdx*-nude mice were anesthetized with Hypnorm (VetaPharma, Leeds, U.K., http://www.vetapharma.co.uk) and Hypnovel (Roche, Welwyn Garden City, U.K., http://www.roche-applied-science.com) and both hind limbs were irradiated with 18 Gy as described previously [45], to deplete the endogenous satellite cell pool. Three days later, *Myf5*^nlacZ/+^ satellite cells expanded for 9 days on either laminin-111 or fibronectin substrate were washed with phosphate buffered saline (PBS) and detached with Accutase StemPro (Invitrogen). Cells were pelleted in proliferation medium, and 1 million were resuspended in approximately 50 μl. The previously irradiated *mdx*-nude mice were anesthetized with isoflurane, and 100,000 satellite cells (∼5 μl) were engrafted into tibialis anterior (TA) muscles using Hamilton syringes. Fibronectin and laminin-expanded cells were engrafted into right and left legs, respectively, as internal control. After 4 weeks, mice were sacrificed and TA muscles harvested, mounted in gum tragacanth, placed in isopentane precooled in liquid nitrogen, before freezing. 7 μm sections were cut at 100 μm intervals throughout the entire muscle, and stained for dystrophin as described below, for the detection of donor-derived fibers, or with X-gal as previously described [46], for the detection of LacZ-positive donor-derived satellite cells. For each engrafted muscle, the section containing the greatest number of *Myf5*^nlacZ/+^ donor-derived satellite cells was found, and the total number of dystrophin^+^ fibers was counted in the corresponding area on the serial section, as done previously [46]. The presence of both dystrophin and *Myf5*^nlacZ/+^ signal confirmed the donor origin of the fibers and excluded the possibility of them being rare revertant host fibers [47].

### Immunostaining of Fibers, Cells, and Cryosections

All wash stages were 3 × 10 minutes in PBS. 7 μm transverse cryosections were cut from gum tragacanth-embedded TA muscle. Samples were fixed in 4% paraformaldehyde for 10 minutes, washed, permeabilized in 0.5% Triton X-100 in PBS for 6 minutes, washed, and blocked in 10% goat serum (Sigma Aldrich) in PBS for 30 minutes. Primary antibodies were applied in blocking buffer for 1 hour as follows: mouse monoclonal anti-Pax7 (1:50; Developmental Studies Hybridoma Bank [DSHB], Iowa city, IA, dshb.biology.uiowa.edu), mouse monoclonal anti-MyoD1 (1:50; Dako, Ely, U.K., http://www.dako.com), rabbit polyclonal anti-MyoD (1:50; Santa Cruz, Santa Cruz, CA, http://www.scbt.com), mouse monoclonal anti-MyoG (1:50; DSHB), mouse monoclonal anti-laminin (1:100; Sigma Aldrich), rabbit polyclonal anti-laminin (1:200; Sigma Aldrich), rabbit anti-fibronectin (1:100; Sigma Aldrich), rat anti-perlecan (1:5,000; Millipore), rabbit anti-collagen IV (1:400; Abcam, Cambridge, U.K., http://www.abcam.com), mouse anti-collagen VI (1:500; Millipore), mouse anti-dystrophin (1:1,000, custom made). After washing, secondary antibodies were applied for 1 hour as follows: conjugated goat anti-mouse IgG-594 or −488, anti-rabbit IgG-488 or −594, or anti-rat IgG-594 (1:200; Invitrogen). Samples were washed and mounted in fluorescent mounting medium with 4′,6-diamidino-2-phenylindole (DAPI). Satellite cell counts on fibers were counted blindly on a minimum of 20 fibers per animal.

## RESULTS

### Freshly Isolated Muscle Fibers from Large^myd^ Mice Contain More Satellite Cells Than Wild-Type, with a Higher Proportion Entered into the Myogenic Program

**Figure 1:**
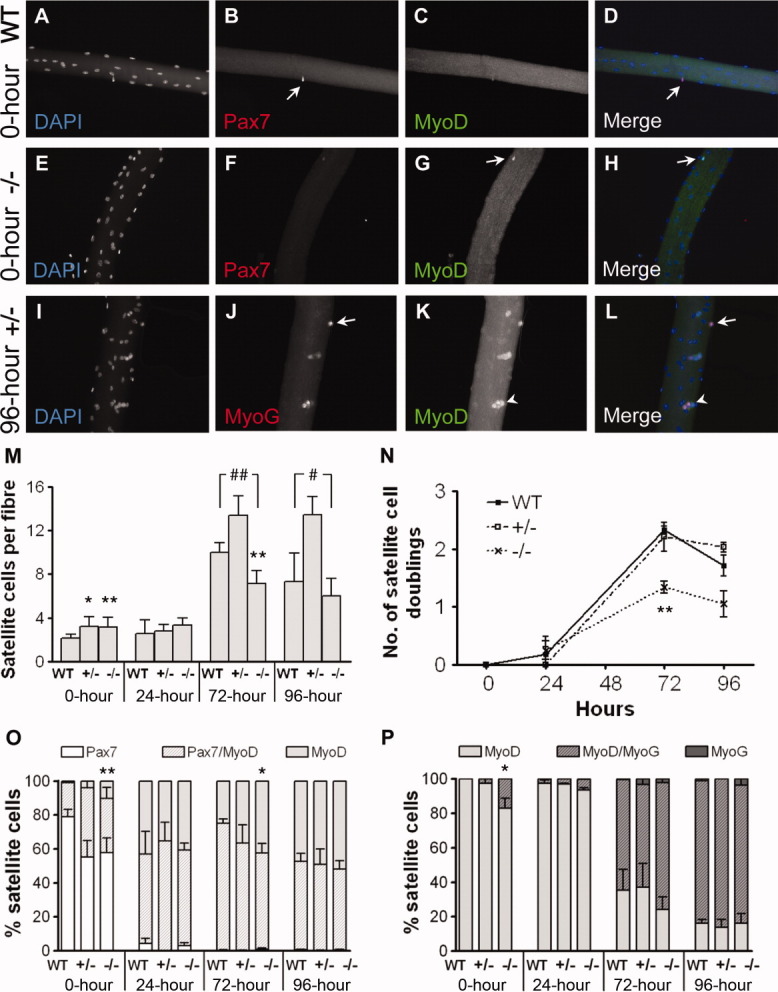
Satellite cells are more abundant and more frequently activated in Large^myd^ muscle but have reduced proliferation compared with wild-type. (A–D): Pax7^+^/MyoD^−^ satellite cell (arrows) on a freshly isolated (0-hour) wild-type fiber. (E–H): Pax7^−^/MyoD^+^ satellite cell (arrows) on Large^myd^^−/−^ fiber (0-hour). (I–L): MyoD^+^/MyoG^+^ (arrows) and MyoD^+^/MyoG^−^ (arrowheads) satellite cells on Large^myd^^+/−^ fiber (96-hour). Magnification ×20. (M): Mean number of satellite cells expressing Pax7 and/or MyoD on wild-type and Large^myd^ single fibers cultured over 96-hour, and corresponding data normalized for mean number of population doublings (relative proliferation) (N). Percentages of Pax7^+^ satellite cells also expressing MyoD (O), and percentages of MyoD^+^ cells also expressing MyoG (P), on wild-type and Large^myd^ single fibers cultured over 96-hour; data from same cells as in graphs (M) and (N). Values are mean ± SEM, *n* = 4–7 animals with ≥20 fibers analyzed per animal. Statistics: Mann–Whitney *U* test. *, significance over wild-type for that time point; #, significance between values. *, *p* <.05; **, *p* <.01. Abbreviations: DAPI, 4′,6-diamidino-2-phenylindole; MyoD, activation marker; MyoG, early differentiation marker; Pax7, quiescence marker; WT, wild type.

In wild-type single fibers from EDL muscles at the time of isolation (0-hour), nuclei expressing the quiescent satellite cell marker Pax7 were observed (Fig. 1A–1D), while in Large^myd^^−/−^ mice, a subset of satellite cells were Pax7^−^/MyoD^+^, an indication of activation/proliferation (Fig. 1E–1H). At 0-hour, Large^myd^^+/−^ and Large^myd^^−/−^ mice had approximately 50% more satellite cells (as assessed by expression of these markers) per fiber than wild-type littermates (Fig. 1M; *p* <.05 and <.01, respectively). In addition, a greater proportion of satellite cells on Large^myd^^+/−^ and Large^myd^^−/−^ single fibers were expressing the activation/proliferation marker MyoD (either with or without Pax7, *p* <.01), and a greater proportion of MyoD^+^ cells were also expressing the early differentiation marker MyoG at 0-hour (*p* <.05), in comparison to wild-type littermates, although this was only significant in Large^myd^^−/−^ mice (Fig. 1O–1P; *p* <.01).

### Satellite Cells on Cultured Large^myd^ Muscle Fibers Display Reduced Proliferation

After isolation, satellite cells on their native fiber become activated, upregulating the expression of MyoD and eventually MyoG, and proliferate. By 96-hour of culture in suspension, the fibers displayed large numbers of MyoD^+^/MyoG^+^ satellite cells/myoblasts (Fig. 1I–1L). Proportions of Pax7/MyoD/MyoG-positive cells per fiber stayed largely similar between Large^myd^ and wild-type mice over 96-hour in culture (Fig. 1O–1P). However, the average number of satellite cells per fiber was significantly greater in Large^myd^^+/−^, compared with wild-type or Large^myd^^−/−^ mice, by 72- and 96-hour (Fig. 1M; *p* <.01 and <.05, respectively). As this does not take into account the differences in initial numbers of satellite cells between the genotypes, the mean number of doublings was calculated from these data; this revealed a similar proliferation rate between wild-type and Large^myd^^+/−^ but a markedly reduced rate in the Large^myd^^−/−^ mice (40% less at 72-hour; Fig. 1N; *p* <.01). We also sought to determine whether satellite cells were undergoing increased apoptosis in the Large^myd^^−/−^ mice, using a terminal deoxynucleotidyl transferase dUTP nick end labeling with bromodeoxyuridine (TUNEL-BrdU) method on single fibers. Only very few nuclei associated with wild-type or Large^myd^^−/−^ fibers were shown to be apoptotic (<1%) at any time point, and these were almost always internal fiber myonuclei (Pax7^−^, MyoD^−^), and there was no significant difference with respect to this parameter between Large^myd^ and control mice (data not shown).

### Proliferation is Restored in Large^myd^ Satellite Cells when Removed from the Environment of Their Parent Fiber

**Figure 2:**
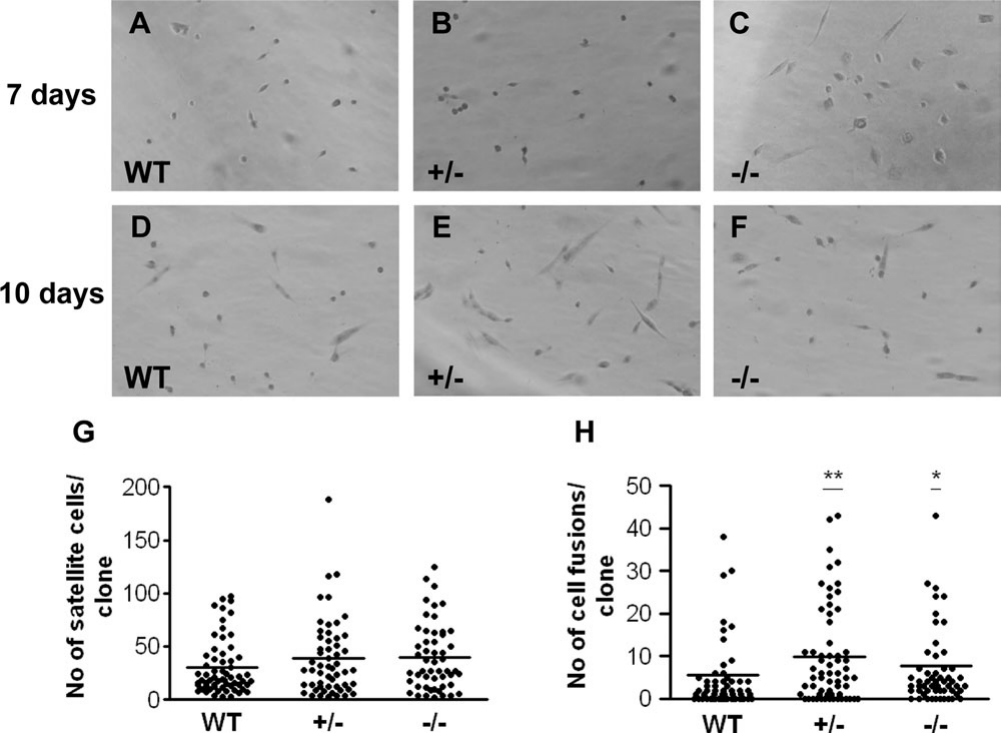
Large^myd^^−/−^ satellite cell proliferation is restored to that of wild type, when removed from the single fiber niche. Single cell-derived satellite cell colonies at 7 days (A–C) or 10 days with a switch to differentiation medium at 7 days (D–F). (A, D), Wild-type cells; (B, E), Large^myd^^+/−^ cells; (C, F), Large^myd^^−/−^ cells. Magnification ×20. Numbers per colony of satellite cells at 7 days (G) and fusion events (myotubes with two or more nuclei) at 10 days (H). Values are individual counts per colony with mean. *n* = 3–4 animals per genotype. Statistics: Mann–Whitney *U* test. *, *p* <.05; **, *p* <.01. Abbreviation: WT, wild type.

In an attempt to determine whether proliferation was impaired in Large^myd^ satellite cells when removed from their native environment, single fibers were stripped of their basal lamina and the released satellite cells collected and cultured on laminin-111 in clonal assays. Surprisingly, at 7 days, there was no significant difference in satellite cell numbers between wild-type, Large^myd^^+/−^, or Large^myd^^−/−^ mice (Fig. 2A–2C, 2G; counts ranged from 3 to 188 per colony). Slight morphological differences were sometimes observed in the Large^myd^^−/−^ cells, apparently resembling a more myoblast/differentiative phenotype than seen in Large^myd^^+/−^ or wild-type mice (Fig. 2A–2C). In addition, fusion was assessed at 10 days, to measure differentiative capacity; Large^myd^^+/−^ and Large^myd^^−/−^ mice displayed a modest but significant increase in the average number of myotubes per colony (Fig. 2D–2F, 2H; *p* <.01 and <.05, respectively; counts ranged from 0 to 43 per colony).

### The Basal Lamina/Interstitium of Large^myd^ Mice Displays an Apparent Increase in Fibronectin Immunolabeling and Fibrosis

**Figure 4:**
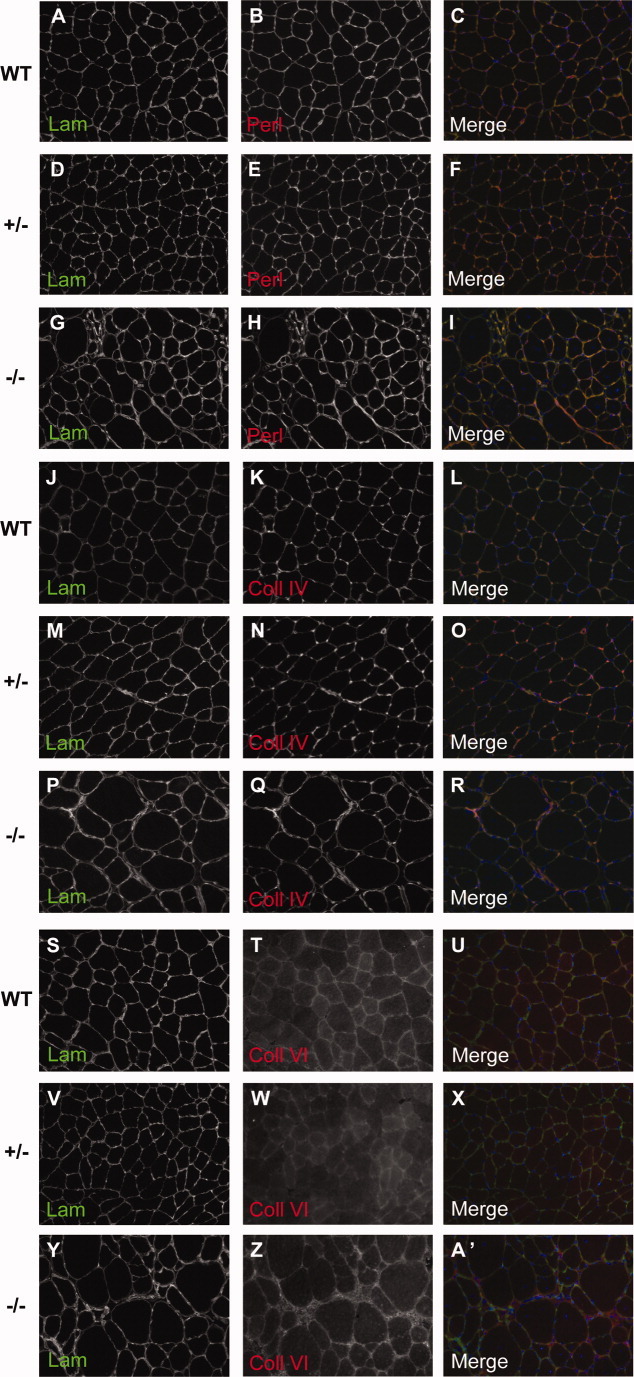
Immunohistochemistry reveals fibrotic Col VI-containing regions in the interstitium of Large^myd^^−/−^ muscle. Basal lamina immunostains of anti-laminin and anti-perlecan in tibialis anterior muscles of wild-type (A–C), Large^myd^^+/−^(D–F) and Large^myd^^−/−^ mice (G–I) at 8 weeks. Basal lamina immunostains of anti-laminin and anti-collagen IV in tibialis anterior muscles of wild-type (J–L), Large^myd^^+/−^(M–O), and Large^myd^^−/−^ mice (P–R) at 8 weeks. Anti-laminin (basal lamina) and anti-collagen VI (reticular lamina/interstitium) immunostain of tibialis anterior muscles of wild-type (S–U), Large^myd^^+/−^(V–X), and Large^myd^^−/−^ mice (Y–A′) at 8 weeks. Magnification ×20. Similar results were observed across three to four animals per genotype. Abbreviations: Col IV, collagen IV; Col VI, collagen VI; Lam, laminin; Perl, perlecan; WT, wild type.

A qualitative analysis of the basal lamina and interstitium was carried out by immunohistochemistry of transverse sections of the TA muscle. A marked increase in fibronectin deposition was observed in Large^myd^^−/−^ (Fig. 3G–3I, 3K), compared to Large^myd^^+/−^ (Fig. 3D–3F) or wild-type (Fig. 3A–3C, 3J) TA muscles. The intensity of the laminin immunolabeling appeared to be unaltered across the three genotypes, and there appeared to be an increase in interstitial space in parts of the Large^myd^^−/−^ muscle (Fig. 3J, 3K). Perlecan (Fig. 4A–4I) and collagen IV (Fig. 4J–4R) appeared at approximately similar intensities across all genotypes. As previously noted, Large^myd^^−/−^, but not Large^myd^^+/−^ mice, display a marked increase in central nucleation (an indicator of past regenerative events) and a greater variability in fiber size relative to wild types, together with deposits of seemingly fibrotic, collagen VI-rich material in the interstitium (Figs. .3I, 3K, 4S–4A′) [41, 44].

**3:**
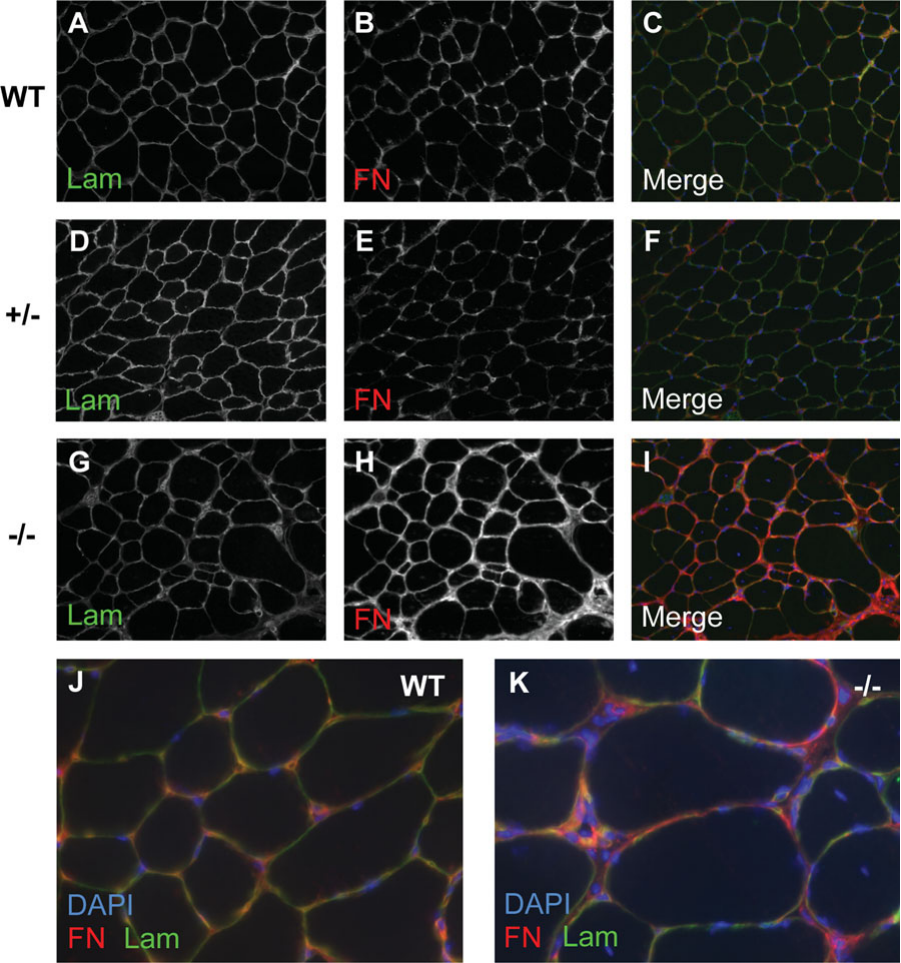
Immunohistochemistry of basal lamina components reveals an upregulation of fibronectin in Large^myd^^−/−^ muscle tissue. Anti-laminin and anti-fibronectin immunostain of tibialis anterior muscles of wild-type (A–C), Large^myd^^+/−^(D–F), and Large^myd^^−/−^ mice (G–I) at 8 weeks. Magnification ×20. (J, K): Merged images at ×40 magnification of wild-type and Large^myd^^−/−^ muscle, respectively. Similar results were observed across three to four animals per genotype. Abbreviations: Lam, laminin; FN, fibronectin; WT, wild type.

### The Satellite Cell Niche of Large^myd^ Muscle Fibers is Often Associated with a Disorganized and Discontinuous Laminin Network

**Figure 5:**
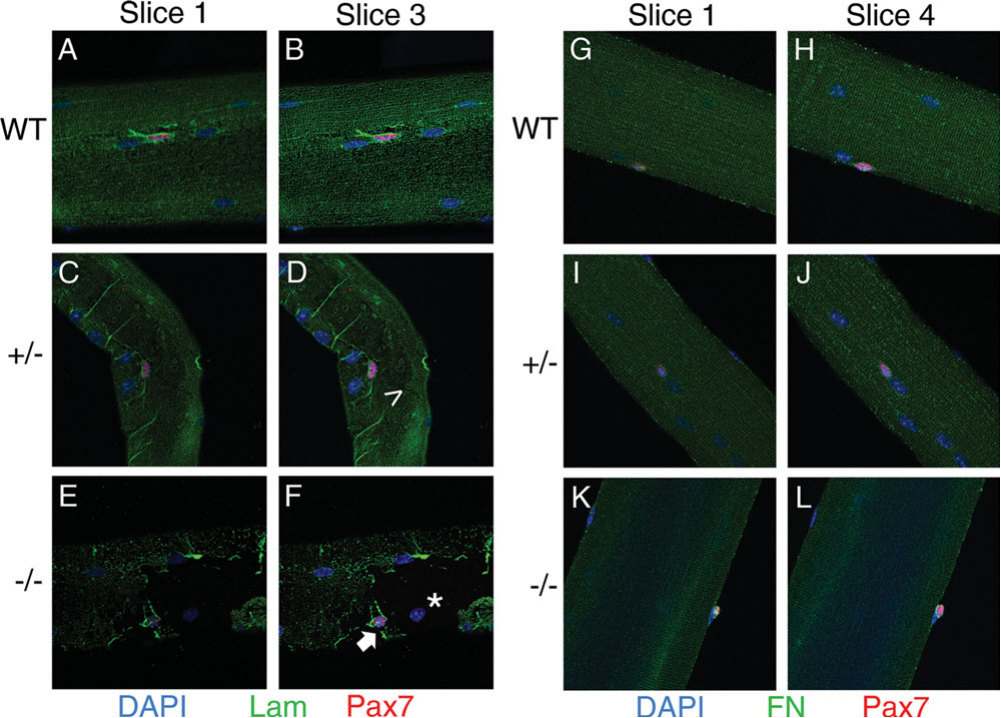
The satellite cell niche in Large^myd^ fibers has a disorganized and discontinuous laminin network but a normal arrangement of fibronectin. Confocal microscope Z-slices of single fibers at different planes. Merged anti-laminin and anti-Pax7 stains in wild-type (A, B), Large^myd^^+/−^(C, D), and Large^myd^^−/−^(E, F) fibers. Large^myd^^+/−^ fibers frequently display small disruptions in the laminin sheath (arrowhead), while Large^myd^^−/−^ fibers display a completely disorganized network, and large regions of discontinuity (*, area with absence of laminin, but an intact fiber underneath complete with myonuclei). Satellite cells can occasionally be observed with incomplete contact with laminin (block arrow). Merged anti-fibronectin and anti-Pax7 stains in wild-type (G, H), Large^myd^^+/−^(I, J), and Large^myd^^−/−^(K, L) fibers. Magnification ×63. Similar results were observed across more than five fibers from two to three animals per genotype. Abbreviations: DAPI, 4′,6-diamidino-2-phenylindole; FN, fibronectin; Lam, laminin; Pax7, quiescence marker; WT, wild type.

In order to obtain a clearer assessment of the satellite cell niche in wild-type and Large^myd^ muscle, and to determine whether the single fiber model retains structural niche integrity, confocal microscopy was employed. Satellite cells appeared to be in contact with a layer of laminin (Fig. 5A–5F) and fibronectin (Fig. 5G–5L) in wild-type, Large^myd^^+/−^, and Large^myd^^−/−^ fibers, as presented here with two separate Z-slices. A layer of fibronectin (observed to be somewhat thicker than the laminin by Z-stack analysis, data not shown) completely covered the basal side of the satellite cell (Fig. 5G, 5I, 5K). Wild-type and Large^myd^^+/−^ fibers had a mesh-like arrangement of laminin (Fig. 5A–5D), with the latter frequently showing small disruptions (arrowhead). In the Large^myd^^−/−^ fibers, the mesh-like arrangement of laminin was markedly disorganized and had more frequent and larger regions of discontinuity than either Large^myd^^+/−^ or wild-type (asterisk, Fig. 5E, 5F). Myonuclei observed in these regions confirmed that the fiber beneath was intact, despite the absence of laminin. Large^myd^^−/−^ satellite cells were observed occasionally to have lost partial contact with the surrounding laminin (block arrow, Fig. 5E, 5F), with laminin absent around parts of the cell, as judged by imaging in more than one plane. Fibronectin appeared to be organized in a similar mesh-like arrangement in both wild-type and Large^myd^ fibers, displaying no disruptions in the latter (Fig. 5G–5L).

### Different Basal Lamina Components have Contrasting Roles in Regulating Satellite Cell Proliferation and Differentiation In Vitro

**Figure 6:**
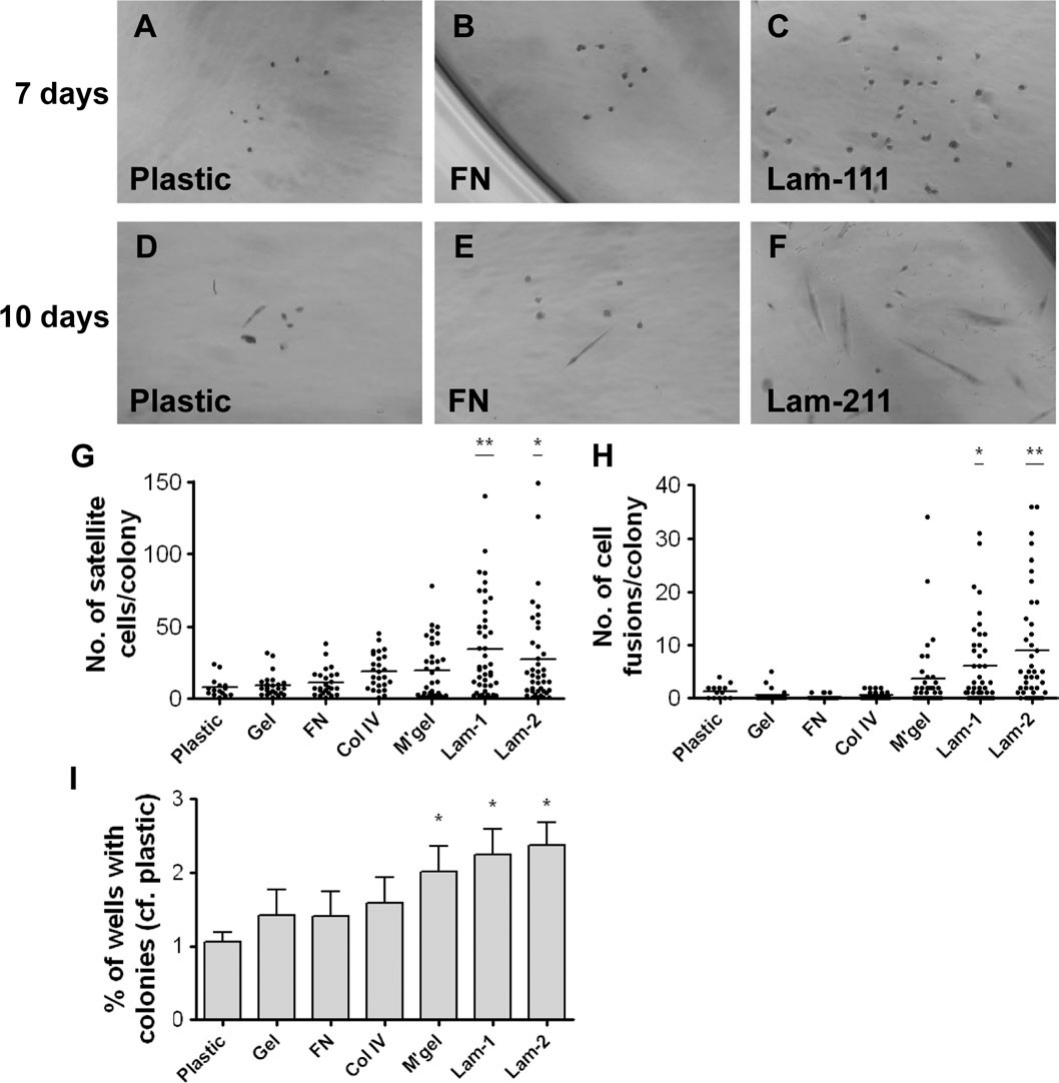
The role of basal lamina components in directing satellite cell proliferation and differentiation. Single cell-derived satellite cell colonies obtained from wild-type mice, at 7 days (A–C) or 10 days with a switch to differentiation medium at 7 days (D–F). Colonies on uncoated plastic (A, D), fibronectin (B, E), laminin-111 (C), and laminin-211 (F). Magnification ×20. Numbers per colony of satellite cells at 7 days (G) and fusion events (myotubes with two or more nuclei) at 10 days (H), *n* = 2 animals per condition, individual counts per colony with mean. Plating efficiency of satellite cells on different substrates compared with uncoated plastic (I), *n* = 4 animals per condition, mean ± SEM. Statistics: Mann–Whitney *U* test. *, *p* <.05; **, *p* <.01. Abbreviation: Col IV, collagen IV; FN, fibronectin; Gel, gelatin; Lam-111, laminin-111; Lam-211, laminin-211; M'gel, Matrigel.

To assess the role of different basal lamina and interstitial components in directing satellite cell function, clonal assays of wild-type mouse satellite cells were carried out on various substrates. Cells plated on plastic, gelatin, fibronectin, and collagen IV made colonies that covered small areas (Fig. 6A, 6B, 6D, 6E) while those plated on laminin-111, −211, and Matrigel migrated so that the colony covered large parts of the well (Fig. 6C, 6F; images show only part of the overall colony). In proliferation studies, average cell number at 7 days was significantly higher on laminin-111 and −211 compared with uncoated plastic (*p* <.01 and <.05, respectively); gelatin, fibronectin, collagen IV, and Matrigel, however, failed to significantly increase proliferation compared with uncoated plastic (Fig. 6G; counts ranged from 3 to 149 per colony). In differentiation studies, the average number of myotubes per colony at day 10 was significantly higher on laminin-111 and −211, compared with plastic (*p* <.05 and <.01, respectively); gelatin, fibronectin, collagen IV, and Matrigel failed to significantly increase differentiation over plastic (Fig. 6H; counts ranged from 0 to 35 per colony). In addition, plating efficiency (percentage of wells that contained colonies, normalized to the value for plastic) was significantly higher on laminin-111, −211, and Matrigel compared with plastic (*p* <.05 for all) but not for gelatin, fibronectin, and collagen IV (Fig. 6I).

### Satellite Cells Expanded on Laminin-111, but not on Fibronectin, Are Able to Contribute Efficiently to Regeneration in Irradiated mdx-nude Mouse Muscle

**Figure 7:**
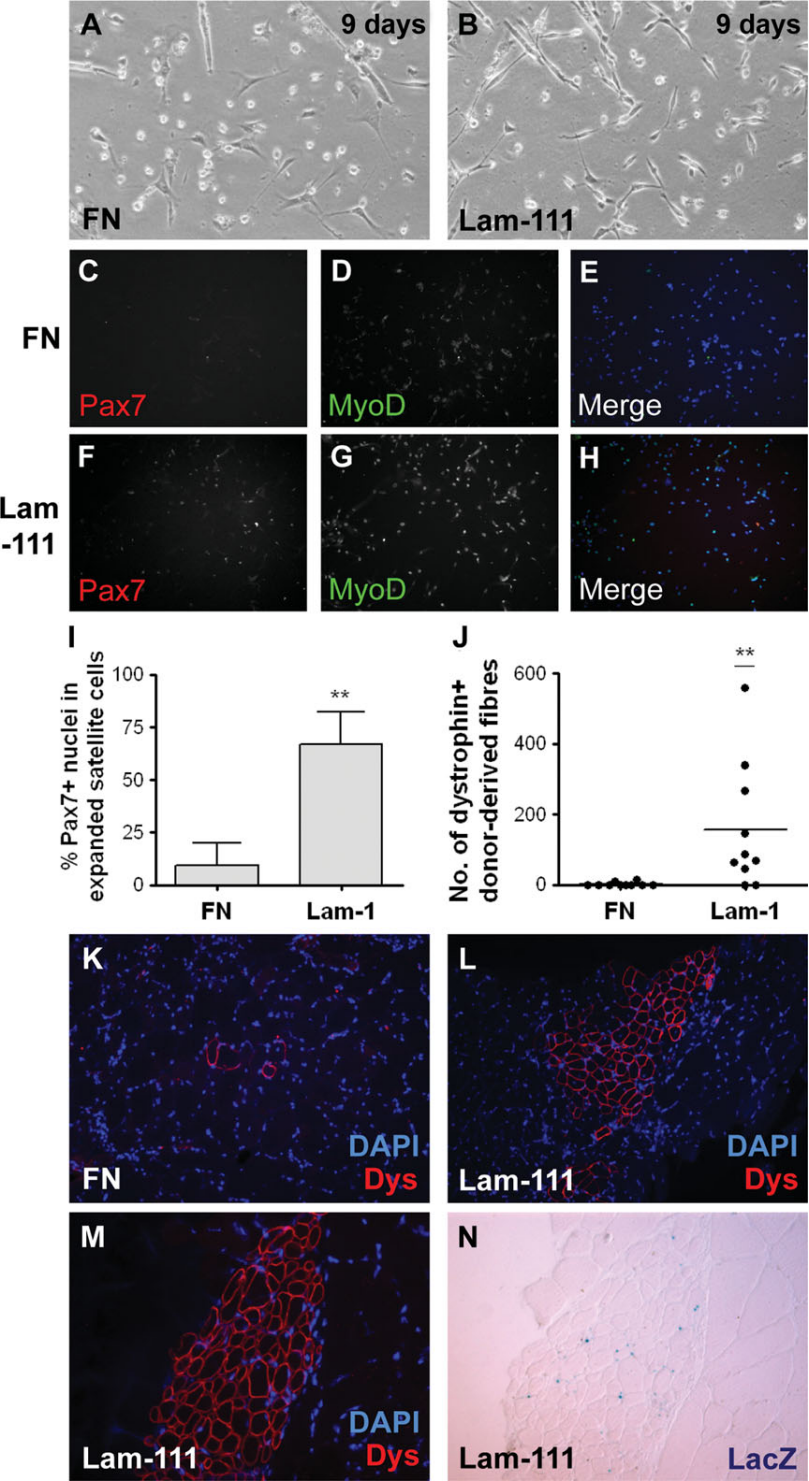
Satellite cells expanded on laminin-111 contribute more efficiently to muscle regeneration in *mdx*-nude hosts than those expanded on fibronectin. *Myf5*^nlacZ/+^ satellite cells/myoblasts expanded for 9 days at 5% oxygen on fibronectin (A) and laminin-111 (B) prior to engraftment into *mdx* host tibialis anterior muscles, ×20 magnification. Corresponding Pax7 and MyoD expression in satellite cells/myoblasts expanded on fibronectin (C–E) and laminin-111 (F–H), ×10 magnification. Percentage of Pax7^+^ nuclei in 9-day donor satellite cells expanded on fibronectin or laminin-111 (I). Mean numbers of dystrophin^+^ fibers derived from donor satellite cells expanded on fibronectin or laminin-111, after engraftment into *mdx*-nude hosts (J). Representative images of dystrophin^+^ fibers derived from donor satellite cells expanded on fibronectin (K) and laminin-111 (L), ×10 magnification. Dystrophin^+^ fibers derived from donor satellite cells expanded on laminin-111 (M), and corresponding area showing donor nuclei expressing lacZ/β-gal (N), ×20 magnification. Values are individual manual counts of dystrophin^+^ fibers (and mean) per section for engrafted tissue (*n* = 10 tibialis anterior muscles), or Pax7-positive cells in wells in an eight-chamber slide for Pax7 expression analysis in expanded cells (*n* = 6–7 wells in an eight-chamber slide, mean ± SEM). Statistics: Mann–Whitney *U* test. **, *p* <.01. Abbreviations: Lam-111, laminin-111; FN, fibronectin; Dys, dystrophin.

To assess whether different basal lamina components are able to influence the in vivo regenerative potential of satellite cells, laminin-111 or fibronectin was used to expand/condition cells, prior to engraftment into irradiated TA muscles of *mdx*-nude hosts. Donor satellite cells from *Myf5*^nlacZ/+^ mice were expanded for 9 days under conditions of low (5%) oxygen and with the addition of 100 ng/ml FGF2, to maximize proliferation. Some differentiation into myotubes occurred on each substrate by 9 days, presumably due to high confluence, but large numbers of unfused myoblasts remained (Fig. 7A, 7B). Mononucleated cells expanded on laminin-111 (Fig. 7F–7H) stained more brightly for MyoD than those on fibronectin (Fig. 7C–7E), and a significantly higher proportion were Pax7^+^ (67.1% compared with 9.51%, *p* <.01, Fig. 7I). When engrafted into the host TA muscle, cells expanded on laminin-111 were able to contribute efficiently to regeneration, as assessed by total number of dystrophin^+^ muscle fibers, compared to cells expanded on fibronectin (Fig. 7J–7L; *p* <.01). Cells expanded on fibronectin gave rise to very few dystrophin^+^ fibers (0–15) , while those expanded on laminin-111 often generated large numbers (mean 158, range 0–559). The donor origin of the dystrophin^+^ fibers was confirmed by the presence of donor-derived *Myf5*^nlacZ/+^ satellite cells or newly formed myonuclei in the same area on serial sections (Fig. 7M, 7N).

## DISCUSSION

Previous investigators have observed altered satellite cell function in various muscular dystrophies, including DMD patients and dystroglycanopathy POMGnT1-null mice [37–40, 48]. In the Large^myd^ mouse, a dystroglycanopathy model with pathology resembling MEB, we report increased numbers (∼50%) of satellite cells in freshly isolated single fibers of Large^myd^^+/−^ and Large^myd^^−/−^ EDL muscles, relative to wild-type (Fig. 1M). In the Large^myd^^−/−^ this most likely reflects the requirement for a larger pool of satellite cells to contribute to the continual regenerative response. Somewhat surprising, however, is the increase observed in the Large^myd^^+/−^ mice, which display no apparent signs of muscle pathology, although it cannot be ruled out that some pathological or environmental changes may be present. Indeed glycosylation of α-dystroglycan has been observed to be slightly reduced in the Large^myd^^+/−^ mice relative to wild-type [49]. Concurrently, a higher proportion of satellite cells express the activation and early differentiation markers MyoD and MyoG, respectively, in Large^myd^^+/−^ and Large^myd^^−/−^ mice (Fig. 1O, 1P) and display increased differentiation in clonal culture (Fig. 2H)‒observations which confirm the recruitment of satellite cells to the regenerative program, due to an ongoing pathology.

In experiments that aimed to elucidate the proliferative potential of satellite cells on cultured intact single fibers (thus maintaining the basic structural elements of the in vivo niche, as evidenced by detection of a network of laminin and fibronectin, Fig. 5), satellite cell proliferation over 96-hour was markedly reduced in Large^myd^^−/−^, but not Large^myd^^+/−^ or wild-type mice (Fig. 1N). However, in in vitro culture after removal from their niche, proliferation and differentiation were restored to that of wild-type (Fig. 2G, 2H). To determine that these effects were a result of an effect on proliferation rather than increased apoptosis, we used TUNEL-BrdU analysis. Apoptosis was very rare in wild-type or Large^myd^ mice at each of the time points (<1% of total fiber nuclei), indicating that our findings are likely to be due to a reduction in satellite cell proliferation and not an increase in their apoptosis (data not shown). These results suggest that the intrinsic regulation of satellite cell proliferation is unaltered by the pathogenic mutation in the Large gene, but that the observed decrease is a direct result of the altered environment caused by the defective glycosylation of α-dystroglycan. This is somewhat in contrast to previous findings by Cohn et al. [50], which suggested that satellite cells also express dystroglycan, and that this is essential for their full regenerative capacity. In this model, a skeletal muscle-specific knockout of dystroglycan (DAG1) mediated by muscle creatine kinase (MCK) Cre recombinase resulted in a surprisingly mild pathology compared with completely null mice; this was attributed to the fact that the MCK promoter is not active in satellite cells, thus allowing them to continue to express dystroglycan. However, some pathological hallmarks were still present here, perhaps indicative of a partially impaired satellite cell pool due to the altered, dystroglycan-null environment [50]. It should also be noted that the consequences of removal of the entire dystroglycan complex could be different to the Large^myd^ mouse, where only glycosylation of α-dystroglycan is altered. In our studies, using the two available antibodies, we were unable to obtain consistent, replicable staining of dystroglycan in satellite cells, either on single fibers or isolated cells (data not shown).

To determine whether there were any alterations in the basal lamina/satellite cell niche of Large^myd^ mouse, we carried out immunohistochemical labeling of muscle sections and single fibers. We observed an upregulation of fibronectin and apparent collagen VI deposits in the interstitium of Large^myd^^−/−^, but not Large^myd^^+/−^ or wild-type sections (Figs. .3A–3K, 4S–4A′). Both are probably an indirect result of muscle pathology, as fibronectin upregulation is seen in regenerating muscle [51] and in other myopathies [52–54], and collagen VI deposition is an indicator of fibrosis [53, 55]. In addition to these observations, there was a severe disorganization and discontinuity of the mesh-like laminin network in Large^myd^^−/−^, but not in Large^myd^^+/−^ or wild-type single fibers, including occasional satellite cells with incomplete contact with laminin (Fig. 5A–5F). The network of fibronectin appeared to be regular and identical in wild-type and Large^myd^ fibers (Fig. 5G–5L), which would suggest that the alterations to laminin are not an artifact of isolation but a direct result of defective dystroglycan glycosylation and binding. As previously mentioned, basal lamina deposition and organization is known to be directed by receptors such as dystroglycan [6, 35].

Given that laminin is known to be an important regulator of mitogenic and regenerative properties of satellite cells [29–31, 36], it seems likely that the alterations in the basal lamina of Large^myd^ mice are a cause for dysregulation of satellite cells. To further investigate this, we examined the behavior of satellite cells on a variety of basal lamina components in vitro. As expected, satellite cells were able to attach, proliferate, and differentiate extensively on laminin-111 and −211 but not on collagens or fibronectin (Fig. 6G–6I) as reported before [36, 56]. Satellite cells expanded for 9 days on laminin-111 (Fig. 7C–7E) stained more brightly for MyoD than those on fibronectin (Fig. 7F–7H) and had a significantly greater proportion expressing Pax7 (67.1% compared with 9.51%, Fig. 7I). Further to this, *Myf5*^nlacZ/+^ donor satellite cells expanded on laminin-111 contributed efficiently to regeneration when engrafted into the irradiated TA muscles of *mdx*-nude hosts (mean 158 dystrophin^+^ fibers, range 0–559), whereas those expanded on fibronectin did not (<15 fibers; Fig. 7J–7L).

The *mdx*-nude host strain is widely used to assess the regenerative potential of engrafted cells, as it is dystrophin^−^, and donor-derived fibers from non-*mdx* animals are dystrophin^+^. To avoid including rare revertant host fibers (that express dystrophin [47]), only dystrophin^+^ fibers where LacZ staining was observed (to denote the presence of donor nuclei), were included in the analysis (Fig. 7M, 7N). It is also worth noting that despite the presence of only a few visible *Myf5*^nlacZ/+^-positive donor nuclei (either satellite cell or recently formed myonuclei), large numbers of dystrophin-expressing fibers were observed, due to the high regenerative potential of the administered satellite cells. While laminin-211, rather than −111, is the major laminin isoform in muscle, the latter binds to the same cell receptors as −211 (integrin α7 and dystroglycan, [4, 57]) and has been previously shown to have similarly positive effects on satellite cell function in vitro (this study and [36, 56]) and in vivo [30], while being more easily and cheaply available. In addition, our in vitro data show that wild-type satellite cells attach, proliferate, and differentiate similarly on laminin-111 and −211 (Fig. 6G–6I).

In this study, we report that satellite cell proliferation is impaired in the Large^myd^^−/−^ mouse, and that this may at least be a partial result of an altered basal lamina microenvironment. The disorganization and discontinuity in the laminin layer (Fig. 5A-5F) may not only influence satellite cell behavior directly but may also lead to direct contact with nonpermissive factors such as fibronectin and/or collagens. It might also be envisaged that the increased deposition of fibronectin and collagen in the interstitium of Large^myd^ muscle (Figs. .3 and 4S–4A′) may further hamper regeneration, when satellite cells migrate from beneath the basal lamina after activation [21, 27]. The observed increase in satellite cell numbers in Large^myd^^+/−^ muscle is unexpected given the lack of obvious pathology, however, a slight reduction in α-dystroglycan glycosylation in the heterozygotes may be responsible [49], and the minor disruptions visible in the laminin in the single fibers of these animals (Fig. 5C, 5D) further suggest slight alterations in the niche and/or fiber instability.

While the structural components of the niche play a major role in satellite cell regulation, in the in vivo setting many more factors are involved. If satellite cells express dystroglycan as is thought [50], then defective glycosylation may directly affect their activity through their adhesion to laminin and thus intracellular signaling. Satellite cell regulation is also mediated by many other interactions, including cues from fibroblasts and from cells in the blood capillary walls [20, 21], and it cannot be excluded that these mechanisms are disrupted in the mutant mice. Previous observations also suggest that satellite cell function may be impaired in the diseased environment, for example, in DMD and in models of dystroglycanopathy [37–40, 48]. Other studies in disease models have pointed to an eventual, cell-intrinsic decline in satellite cell proliferative activity, due to shortened telomeres after multiple rounds of regeneration [32–34], however, it is unclear in these investigations whether there is also a role for an altered environment in their direct dysregulation. The full relationship between niche and satellite cell is still not yet understood, but more studies to further elucidate these interactions would be extremely useful for unraveling the mechanisms of disease.

## CONCLUSION

Satellite cell proliferation was found to be impaired in the Large^myd^ mouse relative to wild-type controls. This was attributed largely to environmental rather than cell-intrinsic effects, as the proliferative activity was restored to that of wild-type, when satellite cells were isolated from their native fiber and expanded on a permissive substrate. Large^myd^ muscle characteristically fails to properly glycosylate α-dystroglycan, and this is associated with an increase in fibronectin, fibrosis and a disorganization of laminin. These compositional changes appear to have a detrimental effect on satellite cell proliferative activity, demonstrating the importance of basal lamina/niche components in the regulation of satellite cell regenerative function.

## Author contributions

J.R.: collection and/or assembly of data, data analysis and interpretation, manuscript writing, and final approval of manuscript; A.B., J.J., and L.B.: collection and/or assembly of data and final approval of manuscript; F.M.: conception and design, financial support, and final approval of manuscript; J.E.H.: conception and design, manuscript writing, and final approval of manuscript; S.C.B. and J.E.M.: conception and design, financial support, data analysis and interpretation, manuscript writing, and final approval of manuscript.
